# Sensor-Based Assessment of Task-Dependent Visual–Postural–Muscular Responses to Smartphone Holder Use During a Simulated Riding-Posture Task

**DOI:** 10.3390/s26113458

**Published:** 2026-05-30

**Authors:** Yi-Lang Chen, Yu-Ju Hung

**Affiliations:** Department of Industrial Engineering and Management, Ming Chi University of Technology, New Taipei 243303, Taiwan; m12218004@mail2.mcut.edu.tw

**Keywords:** scooter-mounted smartphone use, smartphone holder, electromyography, motion analysis, posture, sensor-based ergonomics

## Abstract

Smartphone-holder use during motorcycling is increasingly common, but its task-dependent ergonomic effects remain insufficiently understood. This study examined visual, postural, and muscular responses during smartphone-holder use under a simulated riding-posture condition. Forty healthy adults completed five smartphone-use tasks: dynamic viewing, static viewing, texting, seated use, and standing use. Each riding-related task condition lasted one minute, with the final 30 s designated as the stable data collection window. For postural variables, instantaneous values were recorded at four time points (0, 10, 20, and 30 s from the onset of the stable window) and averaged. For electromyography (EMG), integrated EMG (IEMG) was computed over the same 30 s window using ten consecutive non-overlapping 3 s epochs, and averaged for normalization. The neck flexion (NF), upper thoracic angle (UTA), gaze angle (GA), viewing distance (VD), and electromyographic activities of the cervical erector spinae (CES) and upper trapezius (UTZ) were measured using integrated motion-analysis and EMG approaches. Two-way mixed ANOVA and repeated-measures correlation analyses were performed. The task condition significantly affected all measured variables, with effect sizes ranging from moderate to large (all ηp^2^ ≥ 0.155), with texting producing the greatest NF, shortest VD, and highest muscle activation. Strong within-subject associations were identified among visual, postural, and muscular variables across riding-related tasks (VD–NF: r = −0.815, *p* < 0.001). Females exhibited higher CES and UTZ activation than males. These findings reveal a task-dependent visual–postural–muscular co-variation pattern during scooter-mounted smartphone-holder use and support the application of a sensor-based ergonomic assessment for characterizing task-dependent visual–postural–muscular responses during scooter-mounted smartphone-holder use.

## 1. Introduction

The integration of smartphones into everyday transportation has fundamentally altered human interaction with the environment. In motorcycling, smartphones are increasingly used for navigation, communication, and real-time information access through handlebar-mounted holders. This behavioral shift has introduced new ergonomic and safety challenges, particularly in dynamic riding contexts where visual attention, postural control, and motor coordination must be continuously maintained.

The rapid expansion of app-based delivery services has further amplified this issue; for example, the number of delivery workers in China exceeds 13 million [1]. Delivery riders frequently rely on smartphones during riding to manage routes and tasks under time pressure, resulting in prolonged and repetitive exposure to smartphone use. However, smartphone holder use is not limited to delivery riders; it has become widespread among general motorcyclists, making it a broader human-factors concern.

A substantial body of research has examined the effects of smartphone use on posture and musculoskeletal load in static conditions. These studies consistently report increased neck flexion (NF), forward head posture, and elevated cervical loading during smartphone interaction. For example, biomechanical modeling has shown that cervical spine loading increases progressively with forward head tilt [2,3,4,5,6]. Empirical studies further indicate that smartphone use is associated with sustained non-neutral neck posture and an increased risk of musculoskeletal symptoms in the neck and upper extremities [7,8,9,10]. In addition, cervical flexion during smartphone tasks has been linked to altered spinal alignment and segmental loading, particularly during texting activities [11,12,13].

Previous research has also demonstrated that smartphone use is associated with increased muscle activity in the neck–shoulder region, particularly in the cervical erector spinae (CES) and upper trapezius (UTZ) muscles, due to the need to maintain non-neutral postures [14,15]. Moreover, visual factors such as viewing distance (VD) and screen position have been shown to influence posture and musculoskeletal loading, with a shorter VD leading to greater NF and increased mechanical demand [2,16].

Beyond static conditions, smartphone use in dynamic contexts further challenges postural control and sensorimotor coordination. For example, texting while walking has been shown to alter gait patterns and reduce stability, reflecting the need to manage competing visual and motor demands [17,18]. These findings suggest that mobile device interaction requires the continuous adaptation of visual and postural strategies to maintain task performance.

From a safety perspective, smartphone use has been widely recognized as a major source of visual and cognitive distraction. Off-road glances, reduced gaze stability, and increased cognitive load have been shown to impair situational awareness and increase the crash risk [19,20,21]. Systematic reviews of mobile phone distraction in driving contexts further confirm that such effects are robust across diverse conditions [22]. While these effects are well-established in car driving, their implications for motorcycling remain less understood.

Motorcycling presents unique challenges compared to other forms of transportation. Riders must maintain balance, respond to environmental perturbations, and coordinate visual input with motor output without structural support. These demands require precise sensorimotor integration and may increase the susceptibility to disruption from secondary-task demands [23]. Introducing smartphone interaction into this context may therefore impose additional demands on both visual attention and postural stability.

Despite these concerns, research specifically examining smartphone holder use during motorcycling is limited. Existing studies have largely treated smartphone use as a uniform behavior, without considering the variability introduced by different task demands. In real-world scenarios, smartphone interaction involves multiple task types, such as briefly glancing at notifications, viewing information while stationary, and actively texting. These tasks differ in visual demand, cognitive load, manual involvement, and riding-context constraints, and are therefore likely to produce distinct effects on posture, muscle activity, and visual behavior.

From a human-factors perspective, these adaptations can be conceptualized as task-dependent control strategies, in which individuals dynamically adjust their visual and postural behavior to meet task demands. However, such adaptations may involve trade-offs. For example, reducing the VD may improve visual clarity but increase NF and muscular loading. At the same time, a downward gaze and visual diversion from the forward environment may reduce visual availability for the riding scene and increase potential safety-related concerns.

Furthermore, the interdependence among visual behavior, posture, and muscle activity suggests the presence of a visual–postural–muscular coupling mechanism, in which changes in one domain propagate to others. However, empirical evidence supporting this integrated relationship in motorcycling contexts remains scarce.

Therefore, the present study aims to investigate the effects of smartphone holder use on visual behavior, head–neck posture, and muscle activity during a simulated riding-posture task under different task conditions. Specifically, this study seeks to (1) examine task-dependent changes in posture, visual behavior, and muscle activity; (2) explore sex differences in these responses; and (3) interpret their ergonomic implications for holder placement and interaction design. The present study extends the existing literature in three specific respects. First, whereas prior smartphone ergonomics research has predominantly examined static seating or walking conditions, this study addresses a motorcycling context imposing distinct postural and attentional demands. Second, rather than treating smartphone use as a uniform behavior, this study systematically varies the task type—glancing, viewing, and texting—to characterize task-dependent response patterns. Third, the integration of kinematic, visual, and surface electromyographic measurements within a within-participant repeated-measures correlation framework provides a more comprehensive characterization of visual–postural–muscular interdependence than reported in comparable studies. Recent sensor-based studies have used optical motion capture to quantify the cervical kinematics during mobile-device tasks [24], supporting the relevance of objective kinematic measurement for assessing device-related neck loading. By integrating biomechanical, visual–behavioral, and electromyographic perspectives within a scooter-mounted smartphone-use context, the present study provides a more comprehensive characterization of task-dependent visual–postural–muscular responses than previously reported and offers practical insights for ergonomic design and smartphone-holder placement.

## 2. Materials and Methods

### 2.1. Participants

Forty healthy young adults (20 males and 20 females; age range: 18–30 years) were recruited for this study. All participants were right-handed and held a valid motorcycle license and had prior experience using smartphone holders during riding. Inclusion criteria included normal or corrected-to-normal vision (≥0.8), absence of musculoskeletal disorders, and familiarity with smartphone navigation. All participants provided informed consent prior to participation, and the study procedures were conducted in accordance with institutional ethical guidelines.

### 2.2. Experimental Design

The selection of these five tasks was informed by observational and epidemiological evidence indicating that glancing at notifications, viewing information at traffic stops, and texting while stationary represent the most commonly documented smartphone interaction behaviors among motorcyclists and delivery riders in real-world settings [19,20]. These tasks were further grounded in a human-factors framework characterizing smartphone interactions along dimensions of visual demand and degree of manual involvement, with glancing, passive viewing, and active texting representing qualitatively distinct interaction modes associated with different levels of postural and muscular loading [11,15]. The two baseline conditions (seated and standing smartphone use) were included to provide normative reference values from ordinary smartphone use outside the riding context.

A mixed experimental design was employed with one between-subject factor (sex: male, female) and one within-subject factor (task condition: five levels). The five task conditions were defined as follows: (1) dynamic viewing—participants viewed smartphone notifications while the simulated riding scene was in motion; this condition was intended to represent brief smartphone checking during a dynamic riding-related scenario; (2) static viewing—participants viewed smartphone information while the simulated riding scene represented a temporary stop, such as waiting at a traffic light; (3) texting—participants actively responded to messages while stationary, reflecting a stopped riding scenario rather than active riding; (4) seated baseline—participants used the smartphone in a natural seated posture outside the scooter-riding context; and (5) standing baseline—participants used the smartphone in a natural standing posture outside the scooter-riding context. These tasks were selected to represent different combinations of visual demand, manual interaction, and riding-context constraints, rather than a strict linear hierarchy of workload. It is acknowledged that the three riding-related tasks differ simultaneously in interaction type and environmental context (moving vs. stationary footage), which precludes orthogonal isolation of task demand from motion context. This design reflects ecologically meaningful task–context combinations as they occur in real-world riding scenarios; however, the observed differences cannot be attributed unambiguously to either factor alone.

### 2.3. Posture Measurement

**Figure 1:**
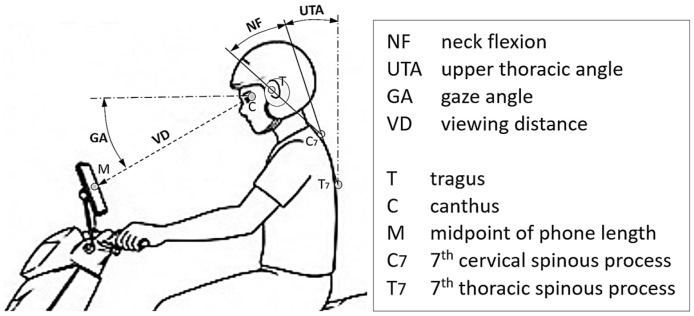
Schematic representation of marker placements and angle definitions for postural measurements.

Postural and visual variables were recorded in the sagittal plane during all five task conditions using a MacReflex motion analysis system (Qualisys, Gothenburg, Sweden) with a sagittal-plane optical tracking setup, operating at a sampling frequency of 60 Hz. System calibration was performed at the beginning of each session following the standard manufacturer procedure. Four reflective markers were attached to anatomical landmarks, including the tragus (T), canthus (C), seventh cervical spinous process (C7), and seventh thoracic spinous process (T7). Because participants wore a helmet during the scooter-simulation tasks, a small opening was created at the corresponding tragus location to ensure accurate marker placement. The helmet was used only to reproduce a riding-related posture and was not intended to provide impact protection during the experiment. Figure 1 illustrates the marker locations and definitions of the measured variables.

The measured variables included NF, upper thoracic angle (UTA), gaze angle (GA), and VD. UTA was defined as the angle between the C7–T7 segment and the vertical reference line. NF represented the relative flexion of the head–neck segment with respect to the upper thoracic segment, thereby accounting for trunk inclination during smartphone use. GA was defined as the angle between the eye-to-smartphone line and the horizontal reference line. VD was measured as the linear distance from the eye region to the midpoint of the smartphone screen, with reference to a wall-mounted scale. These variable definitions were based on previous smartphone posture studies [15,25,26,27].

### 2.4. Surface Electromyography (EMG) Measurement

Surface EMG was used to evaluate muscular activity of the CES and UTZ on the dominant side. EMG signals were collected using a BioRadio system (Great Lakes NeuroTechnologies, Cleveland, OH, USA) equipped with an 8-channel acquisition module. Disposable Ag/AgCl bipolar surface electrodes (10 mm diameter) were attached with an inter-electrode distance of 20 mm. Electrode placement followed recommendations from the SENIAM project [28] and previous neck–shoulder EMG studies [29,30,31,32]. For CES recording, electrodes were positioned approximately 2 cm lateral to the C4 spinous process. For UTZ recording, electrodes were placed midway between the C7 spinous process and the lateral acromion along the muscle fiber direction.

To normalize muscle activity across participants, maximum voluntary contraction (MVC) tests were conducted before the experimental tasks [33]. CES MVC was obtained during resisted neck extension, whereas UTZ MVC was measured during resisted shoulder elevation [30,34]. Each MVC task was repeated three times with sufficient recovery periods between trials to minimize fatigue effects. During each MVC trial, participants sustained maximum isometric contraction for 5 s. After full-wave rectification and smoothing using a 0.5 s moving average window [33], the highest processed EMG value obtained across the three MVC trials was adopted as the normalization reference for each muscle.

Raw EMG signals were bandpass-filtered between 20 and 600 Hz and sampled at 1200 Hz. After full-wave rectification and smoothing using a 0.5 s moving-average window, integrated EMG (IEMG) was computed from the processed EMG signal over each analysis window. IEMG was selected as the primary metric because it reflects cumulative muscle activation over a defined time window and is well-suited for between-condition comparisons within a repeated-measures design. For each experimental condition, the 30 s stable analysis window was divided into ten consecutive non-overlapping 3 s epochs. IEMG was calculated independently for each epoch, and the mean value across epochs was used as the representative muscle activity measure for that condition. Normalization was performed relative to the MVC reference value and expressed as %MVC. Signal quality was visually inspected for each trial; epochs with evident movement artefacts were identified and excluded and replaced using adjacent stable epochs. Kinematic and EMG data collection were initiated simultaneously at the start of each task condition to ensure temporal correspondence between the two measurement modalities.

### 2.5. Experimental Procedure

Upon arrival, participants were briefed on the study procedures and provided written informed consent. Basic demographic information was then collected. Surface EMG electrodes and reflective markers were attached to the predefined anatomical landmarks described above. Before the experimental trials, participants completed maximum voluntary contraction (MVC) tests for EMG normalization.

**Figure 2:**
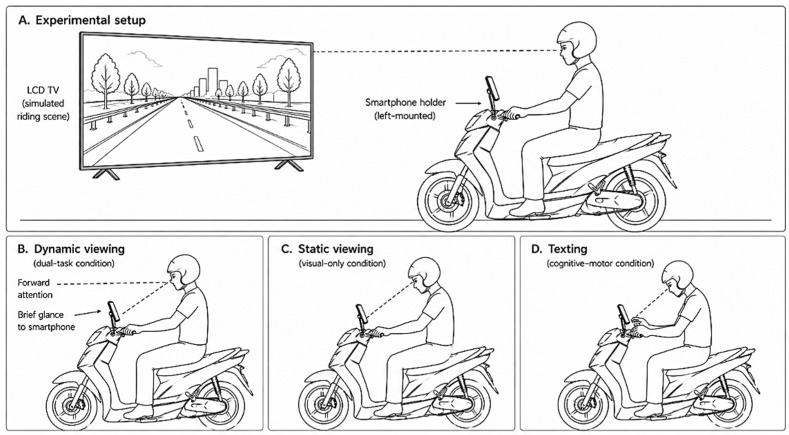
Experimental setup and task conditions. (**A**) Experimental setup showing the participant seated on a scooter with a left-mounted smartphone holder and an LCD TV displaying simulated riding scenes. The gaze direction toward the screen was maintained approximately horizontal to reflect natural forward viewing during riding. (**B**) Dynamic viewing condition: participants briefly glanced at the smartphone while maintaining forward attention. (**C**) Static viewing condition: participants viewed smartphone information while stationary. (**D**) Texting condition: participants actively interacted with the smartphone while stationary, with both hands operating the device mounted on the holder.

A simulated riding-posture setup was used in this study. Participants sat on an electric scooter (Model TS-V7, Taisheng Electric Vehicle, Taoyuan, Taiwan), positioned in front of an LCD screen displaying real-world riding footage (Figure 2A). A smartphone holder was mounted on the left side of the handlebar, reflecting common real-world usage and avoiding interference with throttle control on the right hand. Kinematic data were recorded using a sagittal-plane video setup. Participants were first asked to adjust the smartphone holder to a comfortable and natural viewing position. An LCD displayed pre-recorded riding footage to simulate a forward riding scene. Participants were instructed to maintain a natural riding posture and to look primarily at the simulated road scene, except when the task required them to view or operate the smartphone. The simulated setup was designed to reproduce key postural and visual features of scooter-mounted smartphone use, but it did not reproduce real-road factors such as vibration, traffic flow, or vehicle motion.

Participants used their own smartphones to enhance ecological validity, with the experimental application pre-installed prior to testing. Screen sizes ranged from 5.5 to 6.5 inches, and screen brightness was set according to individual preference, consistent with naturalistic usage. All participants interacted with the smartphone using their dominant hand during smartphone interaction tasks (e.g., texting). The riding-related task sequence consisted of three stages. In the dynamic viewing condition, participants briefly viewed a smartphone notification while the simulated riding footage was playing (Figure 2B). Notifications appeared at randomized intervals, averaging approximately five occurrences per minute, to simulate naturalistic glancing behavior during riding. Participants were instructed to maintain primary attention to the forward riding scene and to glance at the smartphone only when a notification appeared; no manual interaction was required during this condition. Compliance was monitored by the experimenter through direct observation. In the static viewing condition, participants viewed smartphone information while the riding footage represented a temporary stop, such as waiting at a traffic light (Figure 2C). In the texting condition, participants responded to short standardized text messages using the smartphone while stationary; the phone remained fixed on the holder throughout the task (Figure 2D). In the texting condition, both hands were used to interact with the smartphone; it is acknowledged that this differs from actual riding conditions where at least one hand must maintain handlebar contact. The three stages were presented in a fixed sequence to reflect a realistic riding scenario; potential order-related effects are discussed in Section 4.6.

After completing the riding-related tasks, participants performed two baseline smartphone-use conditions for comparison. In the seated baseline, participants used the smartphone in a natural seated posture. In the standing baseline, participants used the smartphone in a natural standing posture. These baseline conditions were used to provide reference values for ordinary smartphone use outside the scooter-riding context.

Participants were instructed to perform all tasks naturally and at a self-selected pace. Short rest periods were provided between trials when needed to reduce fatigue or discomfort. Kinematic and EMG data were collected during each condition, and representative segments from each task were extracted for analysis. For postural variables (NF, UTA, GA, and VD), the final 30 s of each one-minute condition were designated as the stable data collection window. Instantaneous postural values were recorded at four time points at 10 s intervals within this window, and the mean of these four values was used as the representative postural measurement for each condition. For EMG variables, the same 30 s stable window was used for IEMG computation.

### 2.6. Statistical Analysis

All statistical analyses were conducted using SPSS version 23.0 (IBM Corp., Armonk, NY, USA), and the significance level was set at α = 0.05. Descriptive statistics, including means and standard deviations, were calculated for all dependent variables. Prior to the main inferential analyses, assumptions of normality and homogeneity of variance were evaluated using the Shapiro–Wilk test and Levene’s test, respectively. Because the experimental design included five within-subject task conditions and two between-subject sex groups, a two-way mixed analysis of variance (ANOVA) was performed to examine the main and interaction effects of task and sex on all outcome measures (NF, UTA, GA, VD, CES, and UTZ muscle activation expressed as a percentage of MVC (%MVC)). Shapiro–Wilk tests confirmed that normality was satisfied for all dependent variables. Levene’s test indicated homogeneity of variance across sex groups for all variables.

The sphericity assumption for the repeated-measures factor (task condition) was tested using Mauchly’s test. Mauchly’s test was non-significant for all within-subject factors, confirming that the sphericity assumption was met throughout; accordingly, unadjusted degrees of freedom are reported and no Greenhouse–Geisser correction was required. Post hoc pairwise comparisons were conducted using Bonferroni adjustment, and sex main effects were examined using between-group comparisons. Interaction effects were explored via simple effects analyses. Partial eta-squared (ηp^2^) was reported as the measure of effect size and interpreted using conventional benchmarks: small ≥ 0.01, medium ≥ 0.06, and large ≥ 0.14 [35]. Post hoc Bonferroni corrections were applied within each ANOVA; no additional family-wise correction was applied across dependent variables, consistent with standard practice in ergonomics studies examining multiple physiological outcomes within a single experiment.

In addition, repeated-measures correlation analysis was conducted to examine within-subject associations among visual, postural, and muscular variables across the three simulated riding tasks. This method accounts for the non-independence of repeated observations contributed by the same participant and estimates the common within-participant association between two variables. Correlation coefficients (r) and *p*-values were reported. Because seated and standing smartphone-use conditions served as baseline comparison conditions, the repeated-measures correlation analysis focused on the three simulated riding tasks. It is noted that this approach quantifies within-subject associations and does not permit causal inference. Confidence intervals (95%) for all correlation coefficients were computed using the bootstrap method.

## 3. Results

### 3.1. Participant Characteristics

A total of 40 participants (20 males and 20 females) were included in the analysis. The males had a mean height of 173.0 ± 6.4 cm and body weight of 68.4 ± 10.4 kg, whereas the females had a mean height of 161.2 ± 5.0 cm and body weight of 56.8 ± 11.3 kg (Table 1).

### 3.2. Main Effects of Task and Sex

**Figure 3:**
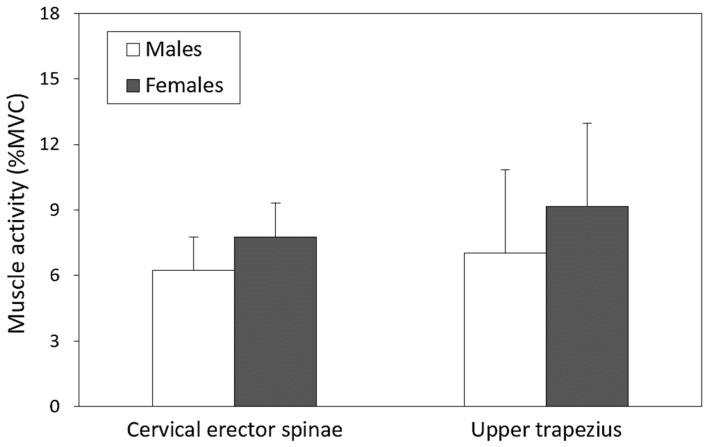
Sex-related differences in cervical erector spinae (CES) and upper trapezius (UTZ) muscle activities across all task conditions. Error bars represent one standard deviation above the mean.

**Figure 4:**
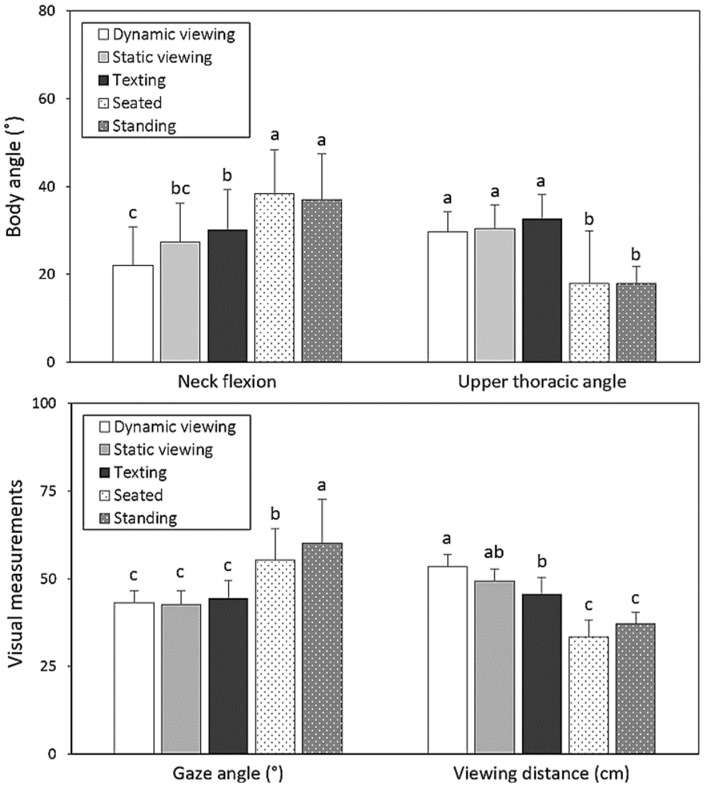
Task-related differences in neck angle and visual measures across the five task conditions. Error bars represent one standard deviation above the mean. Different letters indicate significant differences based on Bonferroni-adjusted pairwise comparisons.

**Figure 5:**
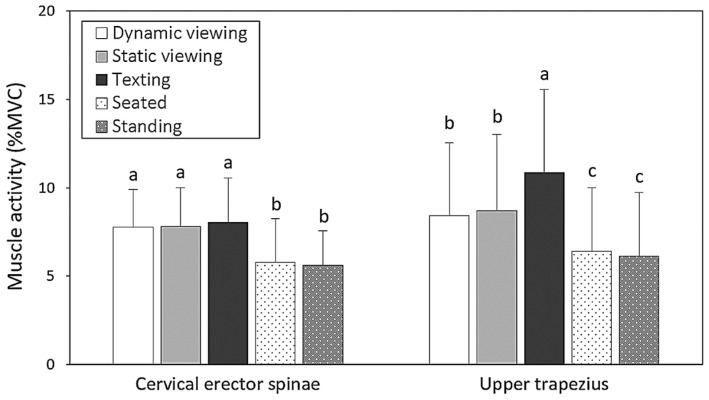
Task-related differences in cervical erector spinae (CES) and upper trapezius (UTZ) muscle activities across the five task conditions. Error bars represent one standard deviation above the mean. Different letters indicate significant differences based on Bonferroni-adjusted pairwise comparisons.

For the overall five-condition comparison, a two-way ANOVA revealed the significant main effects of the task across all dependent variables (*p* < 0.001) (Table 2). Among these, visual variables exhibited the largest effect sizes, particularly the VD (ηp^2^ = 0.691) and GA (ηp^2^ = 0.550), followed by postural variables and muscle activity. For muscle activity, both CES and UTZ showed significant task effects (*p* < 0.001), with moderate effect sizes (ηp^2^ = 0.249 and 0.155, respectively). Postural variables, including NF and UTA, also showed significant task-related variations. Sex effects were observed for CES (*p* < 0.001), UTZ (*p* < 0.001), and the VD (*p* < 0.01), indicating that females exhibited higher muscle activity and a shorter VD than males. Specifically, females showed a shorter VD (41.5 ± 5.3 cm vs. 46.2 ± 5.6 cm in males) and higher CES (7.77 ± 1.6%MVC vs. 6.25 ± 1.5%MVC) and UTZ (9.17 ± 3.8%MVC vs. 7.04 ± 3.8%MVC) activations (Figure 3). The means and standard deviations for postural and visual variables across the five task conditions are presented in Figure 4, whereas Figure 5 presents the corresponding EMG responses. Because the seated and standing conditions served as baseline comparisons, the interpretation of task-specific riding-related responses was further focused on the three scooter-mounted smartphone-use tasks.

### 3.3. Task-Specific Responses Among Riding Conditions

For the focused comparison among the three scooter-mounted smartphone-use tasks (dynamic viewing, static viewing, and texting), distinct patterns were observed. Texting induced the highest UTZ activation, followed by static viewing and dynamic viewing. The CES activity showed a relatively smaller variation across tasks. In terms of posture, NF and UTA increased progressively across these task contexts, with the largest values observed during texting. Visual behavior also varied across task conditions, with texting showing a shorter VD than the other scooter-mounted tasks. Bonferroni-adjusted pairwise comparisons revealed that UTZ activation during texting (10.9 ± 4.7%MVC) was significantly greater than both static viewing (8.7 ± 4.3%MVC) and dynamic viewing (8.4 ± 4.1%MVC), while static and dynamic viewing did not differ significantly from each other. For NF, texting (30.1 ± 9.3°) and static viewing (27.4 ± 8.7°) both produced significantly greater flexion than dynamic viewing (22.0 ± 8.8°). The VD was significantly shorter during texting (45.5 ± 4.9 cm) compared with dynamic viewing (53.4 ± 3.4 cm). Detailed pairwise comparisons are presented in Figure 4 and Figure 5.

### 3.4. Interaction Effects

**Figure 6:**
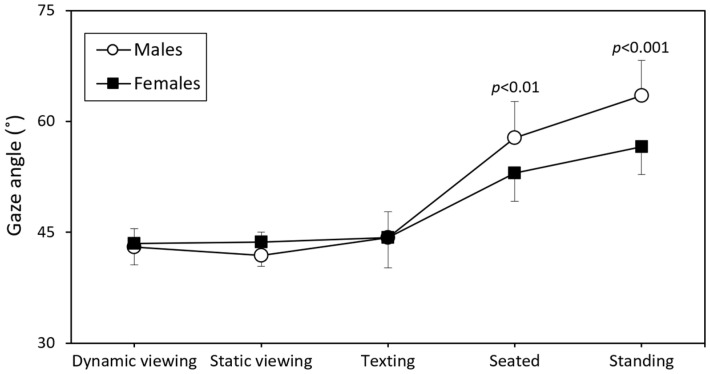
Sex-specific comparisons of gaze angle (GA) across the three riding-related tasks and two baseline conditions. Error bars indicate either +1 or −1 standard deviation to reduce overlap between groups. Significant sex × task interaction effects were observed only for GA.

A significant interaction between sex and task was observed for GA (*p* < 0.05), suggesting sex-specific visual strategies across tasks (Table 2). No other significant interactions were found. Simple effects analyses indicated that the females demonstrated a greater downward GA than the males particularly during the seated (*p* < 0.01) and standing condition (*p* < 0.001), whereas the sex difference in GA was less pronounced in all simulated tasks (Figure 6).

### 3.5. Repeated-Measures Correlations Among Variables

Repeated-measures correlation analysis was conducted across the three riding-related tasks (Table 3). The VD was strongly and inversely associated with NF (r = −0.815, *p* < 0.001), indicating that a shorter VD was linked to an increased forward head posture. The VD was also moderately and negatively associated with UTZ activity (r = −0.548, *p* < 0.001). NF showed a moderate positive association with UTZ (r = 0.449, *p* < 0.001), suggesting that increased NF corresponded to greater shoulder muscle activation. UTA also demonstrated a moderate positive association with UTZ (r = 0.384, *p* < 0.001). In contrast, CES activity showed weaker and non-significant associations with postural variables, indicating a different activation pattern compared to UTZ.

## 4. Discussion

### 4.1. Task-Dependent Control Strategies

The present study demonstrates that smartphone use during the scooter-mounted riding-posture task is strongly task-dependent. Among the three riding-related tasks, texting likely involved greater combined visual, cognitive, and manual demands, resulting in the greatest deviations in posture, visual behavior, and muscle activity. Dynamic viewing, in contrast, involved intermittent glances while maintaining forward attention, resulting in smaller postural changes. These findings support the concept of task-dependent control strategies, in which users adapt their behavior according to task demands.

The observed task-dependent adaptations are consistent with previous research showing that smartphone interaction in dynamic environments requires continuous coordination between visual attention and motor control. For example, Schabrun et al. [17] reported that texting while walking altered gait behavior and postural control strategies as individuals attempted to maintain both locomotor stability and smartphone-task performance. Similarly, Bruyneel et al. [28] concluded that mobile-phone interaction during walking consistently affects gait-related control across different populations.

However, compared with walking-based smartphone studies, the simulated riding-posture context examined here likely imposed additional constraints reflecting real-world motorcycling demands. During actual motorcycle riding, users must simultaneously maintain vehicle control, body balance, environmental monitoring, and smartphone interaction. Unlike walking, where lower-limb locomotion remains relatively automatic, motorcycling requires continuous upper-body stabilization and sensorimotor coordination [23]. Although the present setup did not reproduce all real-world riding dynamics, the postural geometry and visual task demands were designed to reflect scooter-mounted smartphone use, and these combined demands may explain why posture, viewing behavior, and muscle activity changed systematically across task conditions.

From a human-factors perspective, the findings suggest that users dynamically modified their viewing strategy and posture according to the task requirements. More visually demanding tasks, such as texting, appeared to promote a shorter VD and greater NF in order to facilitate visual acquisition and manual interaction. Although such adaptations may improve task performance, they may simultaneously increase biomechanical loading and potentially reduce visual availability for the forward riding environment.

### 4.2. Visual–Postural–Muscular Coupling

A key contribution of this study is the identification of a strong and systematic co-variation among visual behavior, posture, and muscle activity during smartphone-holder use in a simulated riding-posture context. The repeated-measures correlation analysis revealed strong within-subject associations between VD and NF activity (r = −0.815, *p* < 0.001), as well as between VD and UTZ activity (r = −0.548, *p* < 0.001). These findings reflect within-subject associations rather than demonstrated causal mechanisms. Nevertheless, the observed associations suggest that visual adjustments were consistently accompanied by corresponding changes in head–neck posture and muscular loading, consistent with a coordinated visual–postural–muscular co-variation pattern.

This finding is highly consistent with previous smartphone ergonomics studies demonstrating that a shorter VD generally requires greater visual accommodation and is commonly associated with increased cervical flexion [2,16]. Previous laboratory-based investigations have similarly shown that smartphone tasks involving a closer VD or greater visual concentration tend to increase forward head posture and cervical loading [11,14,36]. The increased NF observed during smartphone interaction has also been linked to elevated compressive and mechanical loading on cervical structures [3,6].

Compared with previous smartphone ergonomics studies that primarily examined posture or muscle activity under static or non-riding conditions [11,14,16,36], the present study extends the existing knowledge in two important ways. First, the findings demonstrate that visual–postural adaptations occur dynamically across different riding-related smartphone tasks. Second, the observed associations suggest that visual adjustments were accompanied by changes in head–neck posture and muscular loading.

More specifically, a reduced VD was associated not only with an increased NF but also with elevated UTZ activity, while NF itself showed a moderate positive association with UTZ activation. These associations are consistent with a pattern in which visual demands co-vary with the viewing strategy, head–neck posture, and muscular loading, although the directionality of these relationships cannot be established from the present correlational data.

The strength of the VD–NF association observed in the present study (r = −0.815) further suggests that the viewing behavior may be strongly associated with posture during smartphone-holder use. This finding has practical ergonomic implications because interventions targeting viewing behavior—such as optimizing smartphone-holder placement or improving display readability—may indirectly reduce NF and the associated muscular demand. The robustness of this estimate is supported by the large sample size (n = 40, 120 observation pairs), the use of the rmcorr method which removes the between-subject variance prior to estimation, and the narrow 95% bootstrap confidence interval reported in Table 3.

### 4.3. Muscle Loading Mechanisms and Sex-Related Differences

The results further indicate that UTZ activity is more responsive to task-related changes than CES activity. While both muscle groups contribute to head–neck stabilization, the moderate association between NF and UTZ suggests that UTZ plays a more prominent role in accommodating task-dependent posture changes. One possible explanation is that UTZ is involved not only in head stabilization but also in shoulder girdle control during upper limb activity. Texting requires fine motor control and sustained upper limb positioning, which may increase the demand for scapular stabilization and consequently elevate UTZ activity. This interpretation is consistent with previous findings that UTZ activity increases with both NF and upper limb involvement [14,36]. The comparatively smaller effect sizes for EMG variables (CES: ηp^2^ = 0.249; UTZ: ηp^2^ = 0.155) relative to visual and postural variables (VD: ηp^2^ = 0.691; GA: ηp^2^ = 0.550) reflect the hierarchical responsiveness of these measurement domains: visual and postural variables represent direct, goal-directed behavioral adaptations to task demands, whereas muscle activity is a more distal consequence additionally subject to individual variation in recruitment strategy and co-contraction patterns. EMG signal variability and normalization procedures may further attenuate the EMG effect size estimates relative to kinematic measures.

In contrast, CES activity showed weaker associations with postural variables, suggesting that cervical extensor muscles may respond more to the overall head load rather than fine adjustments in task-dependent posture. This distinction highlights the importance of considering muscle-specific roles when evaluating ergonomic risks.

Sex-related differences further support the need to consider relative muscular demand in smartphone-holder use. Females showed higher CES and UTZ activation and a shorter VD than males. One plausible hypothesis is that these differences may reflect anthropometric factors such as a shorter arm length and lower absolute cervical muscle strength; however, these factors were not directly measured and therefore warrant further empirical confirmation. An alternative hypothesis is that sex differences may reflect behavioral factors, including differences in smartphone interaction strategy, grip posture, or habitual viewing distance beyond anthropometric characteristics. Both explanations remain speculative and await direct empirical testing. Similar sex- and posture-related differences in neck muscle activity during smartphone tasks have been reported previously [15,34]. However, because significant sex × task interactions were generally absent, except for GA, the overall task-dependent response pattern appeared broadly similar between males and females. This suggests that task demand was the primary driver of visual–postural–muscular responses, while sex-related factors may modulate the magnitude of muscular loading. Future studies should include direct measurements of anthropometric variables and maximal cervical muscle strength to better characterize the mechanisms underlying sex-related differences in neck–shoulder loading.

### 4.4. Safety and Human-Factors Implications

The following interpretations are indirect inferences derived from the biomechanical and visual data collected in the present study. Hazard perception, situational awareness, reaction time, and vehicle control performance were not measured and cannot be directly inferred from these data. The findings may have safety-related implications for scooter-mounted smartphone-holder use, but these implications should be interpreted cautiously. The association between visual behavior and posture suggests that changes in viewing strategy, such as reducing the VD, may be accompanied by meaningful biomechanical changes. In addition, a downward gaze during smartphone use may reduce visual availability for the forward scene. When combined with the cognitive and manual demands of texting, this pattern may represent potential human factors concerns that warrant further investigation using direct measures of hazard detection, reaction time, and riding performance. The increased muscle activity observed during higher-demand tasks may, over prolonged real-world exposure, contribute to fatigue accumulation; however, as no fatigue measures were collected in the present short-duration laboratory study, this remains speculative. This is particularly relevant for high-exposure users such as delivery riders. It should also be noted that the present study examined smartphone-holder use under controlled laboratory conditions for the purpose of ergonomic risk characterization, and does not in any way endorse or encourage smartphone interaction during actual motorcycle or scooter operation.

### 4.5. Practical Implications

The findings suggest that reducing the viewing demand and maintaining a greater VD may help minimize the biomechanical load. Smartphone holder placement should therefore aim to avoid an excessive downward gaze and reduce NF, preferably by positioning the display closer to the rider’s natural forward line of sight without obstructing road visibility. Interfaces intended for motorcycle use should support rapid information acquisition with minimal interaction time, such as simplified layouts, larger text, high-contrast displays, and voice- or audio-based guidance. High-demand tasks such as texting should be avoided during riding-related use, and design or platform-level restrictions may be considered to discourage manual interaction while riding. From a sensor-based ergonomics perspective, the combined use of kinematic and EMG measurements also provides a practical framework for identifying task conditions that may increase visual and musculoskeletal demand, consistent with recent work using wearable sensors and surface EMG to monitor posture and muscle activation for ergonomic risk assessment [37].

### 4.6. Limitations and Future Research

Several limitations should be acknowledged. First, the simulated riding environment did not reproduce real-world conditions such as road vibration, active traffic, or vehicle motion. However, controlled simulation is a well-established methodology in ergonomics and human-factors research, and has been adopted in comparable studies examining posture and muscle activity during device use in vehicle-related contexts [15,27]. The present simulation was designed to reproduce the postural geometry and visual task demands of scooter-mounted smartphone use, which are the primary determinants of the biomechanical outcomes measured. The absence of dynamic road inputs may have reduced the overall physical demand relative to real riding; however, this conservative condition would more likely underestimate rather than inflate the ergonomic effects observed, lending support to the directional validity of the findings. The observed visual–postural–muscular coupling patterns may therefore be relevant to real-world riding contexts, while safety-related interpretations involving situational awareness or riding performance await confirmation through on-road studies. The terminology used throughout the manuscript has been revised accordingly to reflect that the findings pertain to a simulated riding-posture task rather than actual motorcycling.

Second, the riding-related tasks were presented in a fixed sequence to reflect a realistic riding scenario. However, the absence of counterbalancing may have introduced order-related confounds, including learning, postural adaptation, anticipation, or fatigue accumulation across conditions. Therefore, part of the observed task differences may partially reflect sequence-related adaptation or fatigue effects rather than the task demand alone. Future studies should employ counterbalanced task orders or time-course analyses to better isolate task-specific responses from potential order-related effects.

Third, MVC measurements were collected only prior to the experimental tasks. Given the short task duration and generally low-to-moderate %MVC values observed, a meaningful fatigue-induced MVC decline within the session is considered unlikely; however, future studies should collect both pre- and post-task MVCs to directly verify the normalization stability. Fourth, participants used their own smartphones, introducing variability in the device size (5.5–6.5 inches), weight, and display characteristics; future studies should standardize device specifications to improve reproducibility. Fifth, the texting condition permitted bilateral hand engagement, which does not replicate real-world riding where at least one hand must maintain handlebar contact; future studies should examine single-hand texting configurations. Sixth, the tasks were of a short duration (one minute per condition), which may underestimate the cumulative musculoskeletal loading under prolonged real-world exposure; future studies should use longer exposure protocols with validated fatigue outcome measures. Seventh, no direct anthropometric or physiological measurements (e.g., arm length, and cervical muscle strength) were collected, limiting the interpretation of the observed sex-related differences. Finally, the smartphone holder was fixed in a single position; future studies should examine different mounting configurations and heights. The sample comprised healthy young adults aged 18–30, which may not represent older riders or delivery workers with greater habitual exposure and potentially different anthropometric and muscular profiles.

## 5. Conclusions

This study examined task-dependent visual, postural, and muscular responses during smartphone-holder use in a simulated riding-posture task. Among the five task conditions, texting produced the greatest neck flexion, shortest viewing distance, and highest muscle activation, while baseline seated and standing conditions resulted in more favorable postural profiles. These findings confirm that the smartphone interaction type substantially influences the biomechanical demands imposed during scooter-mounted smartphone use.

A central finding was the strong association between a shorter VD and greater NF, together with moderate associations between VD, NF, and UTZ activity. These results suggest a visual–postural–muscular coupling mechanism whereby users adjust their visual strategy to meet task demands, although such adjustments may increase biomechanical loading.

From a human-factors perspective, smartphone-holder use may introduce ergonomic challenges by promoting a downward gaze, altering visual behavior, and increasing the neck–shoulder muscular demand. The potential safety implications associated with these changes require further investigation using direct riding-performance measures. It should be emphasized that the present study was conducted under controlled laboratory conditions for the purpose of ergonomic risk characterization, and does not endorse smartphone interaction during actual riding. Although delivery riders motivated the present study, the findings are relevant to general motorcyclists who use smartphone holders for navigation or communication. Future design and usage guidelines should consider the holder placement, viewing distance, interface simplicity, and avoidance of high-demand interactions such as texting during riding. These results also support the potential value of sensor-based monitoring approaches for evaluating ergonomic risks in scooter-mounted smartphone-holder use.

## Figures and Tables

**Table 1:** Descriptive information (mean ± standard deviation) of male and female participants.

Items	Males (n = 20)	Females (n = 20)
Age (years)	21.5 ± 1.6	22.0 ± 1.4
Stature (cm)	173.0 ± 6.4	161.2 ± 5.0
Body mass (kg)	68.4 ± 10.4	56.8 ± 11.3

**Table 2:** Two-way ANOVA results for all measured responses.

Variables	Responses	F	*p*-Value	ηp^2^
Sex	Neck flexion	3.13	0.079	0.076
	Upper thoracic angle	1.95	0.164	0.049
	Gaze angle	3.18	0.076	0.077
	Viewing distance	8.33	<0.01	0.175
	Cervical erector spinae	25.08	<0.001	0.398
	Upper trapezius	13.85	<0.001	0.239
Task	Neck flexion	19.58	<0.001	0.340
	Upper thoracic angle	40.08	<0.001	0.513
	Gaze angle	46.46	<0.001	0.550
	Viewing distance	84.99	<0.001	0.691
	Cervical erector spinae	12.61	<0.001	0.249
	Upper trapezius	6.99	<0.001	0.155
Sex × Task	Neck flexion	0.17	0.956	0.004
	Upper thoracic angle	0.27	0.896	0.007
	Gaze angle	2.46	<0.05	0.061
	Viewing distance	1.14	0.340	0.029
	Cervical erector spinae	0.37	0.828	0.010
	Upper trapezius	0.05	0.996	0.001

**Table 3:** Repeated-measures correlations among task-related variables across the three simulated riding tasks.

Variable Pair	r	95% CI	*p*-Value
NF–CES EMG	0.119	−0.102–0.333	0.289
NF–UTZ EMG	0.449	0.253–0.607	<0.001
UTA–CES EMG	0.090	−0.117–0.288	0.426
UTA–UTZ EMG	0.384	0.179–0.559	<0.001
VD–NF	−0.815	−0.890–−0.702	<0.001
VD–GA	−0.095	−0.304–0.113	0.400
VD–CES	−0.171	−0.371–0.048	0.126
VD–UTZ	−0.548	−0.685–−0.362	<0.001

Note: NF, neck flexion; UTA, upper thoracic angle; GA, gaze angle; VD, viewing distance; CES, cervical erector spinae; UTZ, upper trapezius; EMG, electromyography; CI, confidence interval.

## Data Availability

The datasets generated and analyzed during the current study are available from the corresponding author upon reasonable request.

## Abbreviations

The following abbreviations are used in this manuscript:

NF	Neck flexion
UTA	Upper thoracic angle
GA	Gaze angle
VD	Viewing distance
CES	Cervical erector spinae
UTZ	Upper trapezius
EMG	Electromyography
ANOVA	Analysis of variance
